# Reduced oxygen saturation entropy is associated with poor prognosis in critically ill patients with sepsis

**DOI:** 10.14814/phy2.15546

**Published:** 2022-12-21

**Authors:** Margaret Gheorghita, Matthew Wikner, Anika Cawthorn, Tope Oyelade, Kristof Nemeth, Patrick Rockenschaub, Ferran Gonzalez Hernandez, Nel Swanepoel, Watjana Lilaonitkul, Ali R. Mani

**Affiliations:** ^1^ Network Physiology Lab, Division of Medicine, UCL London UK; ^2^ Department of Perioperative Medicine and Pain Barts Health NHS Trust London UK; ^3^ ARC Research Software Development Group, Advanced Research Computing, UCL London UK; ^4^ Department of Surgery Queen Elizabeth Hospital London UK; ^5^ Institute of Health Informatics, UCL London UK; ^6^ CoMPLEX, UCL London UK; ^7^ Health Data Research UK London UK

**Keywords:** entropy, oxygen saturation, sepsis, SpO_2_, survival, variability

## Abstract

Recent studies have found that oxygen saturation (SpO_2_) variability analysis has potential for noninvasive assessment of the functional connectivity of cardiorespiratory control systems during hypoxia. Patients with sepsis have suboptimal tissue oxygenation and impaired organ system connectivity. Our objective with this report was to highlight the potential use for SpO_2_ variability analysis in predicting intensive care survival in patients with sepsis. MIMIC‐III clinical data of 164 adults meeting Sepsis‐3 criteria and with 30 min of SpO_2_ and respiratory rate data were analyzed. The complexity of SpO_2_ signals was measured through various entropy calculations such as sample entropy and multiscale entropy analysis. The sequential organ failure assessment (SOFA) score was calculated to assess the severity of sepsis and multiorgan failure. While the standard deviation of SpO_2_ signals was comparable in the non‐survivor and survivor groups, non‐survivors had significantly lower SpO_2_ entropy than those who survived their ICU stay (0.107 ± 0.084 vs. 0.070 ± 0.083, *p* < 0.05). According to Cox regression analysis, higher SpO_2_ entropy was a predictor of survival in patients with sepsis. Multivariate analysis also showed that the prognostic value of SpO_2_ entropy was independent of other covariates such as age, SOFA score, mean SpO_2_, and ventilation status. When SpO_2_ entropy was combined with mean SpO_2_, the composite had a significantly higher performance in prediction of survival. Analysis of SpO_2_ entropy can predict patient outcome, and when combined with SpO_2_ mean, can provide improved prognostic information. The prognostic power is on par with the SOFA score. This analysis can easily be incorporated into current ICU practice and has potential for noninvasive assessment of critically ill patients.

## Graphical abstract

Question: What is the prognostic value of SpO_2_ fluctuations in critically ill patients with sepsis? Findings: SpO_2_ entropy predicts survival in sepsis independent of SOFA score and ventilation status. Meanings: Greater irregularity of SpO_2_ fluctuations is associated with better prognosis in patients with sepsis.

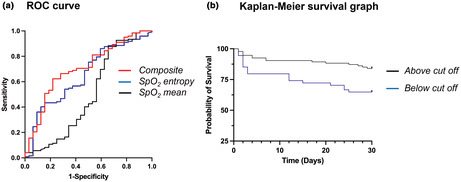

## 1 INTRODUCTION

Pulse oximetry is extensively used in the ICU to monitor cardiorespiratory status and predict hypoxemia. However, as a continuous reading, oxygen saturation (SpO_2_) variability analysis has scarcely been studied for clinical applications. SpO_2_ signals exhibit a complex, fractal‐like pattern in hypoxic individuals (Costello et al., 2020), and information embedded in SpO_2_ fluctuations can be quantified using entropy measures (Bhogal & Mani, 2017). There is mounting evidence that this form of measurement carries information on physiological engagement of the cardiorespiratory system as well as dynamic interaction between oxygen and hemoglobin (Bhogal & Mani, 2017; Jiang et al., 2021; Roe & Jones, 1993). SpO_2_ entropy has been found to have a negative relationship with SpO_2_ mean; however, merely analyzing the mean does not yield sufficient information about the dynamics of the cardiorespiratory system (Bhogal & Mani, 2017; Costello et al., 2020). In graded normobaric hypoxia, as the concentration of inspired oxygen decreases, SpO_2_ entropy increases in healthy individuals. Furthermore, SpO_2_ entropy, but not absolute or mean SpO_2_, is correlated with perception of breathlessness during experimental hypoxia (Costello et al., 2020). These observations suggest that as oxygen becomes scarcer, the physiological system is more engaged and there is a higher transfer of information between SpO_2_ and other respiratory variables (i.e., tidal volume, minute ventilation, respiratory rate, P_ET_O_2_, and P_ET_CO_2_) (Jiang et al., 2021). Within the context of disease, assessing the SpO_2_ entropy in patients with COPD can distinguish and predict stable and exacerbation phases of disease (Al Rajeh et al., 2021).

Sepsis, an inappropriate host response to infection, places an extremely heavy burden on hospitals across the globe, accounting for a staggering ~20% of global deaths in 2017 (Rudd et al., 2020). Currently, the status of patients with sepsis is assessed using the Sequential Organ Failure Assessment (SOFA) score which reflects development of multiorgan dysfunction in this critical illness. Patients with sepsis and septic shock have suboptimal oxygen delivery (Tuchschmidt et al., 1991) and impaired organ system network connectivity (Asada et al., 2016). SpO_2_ variability analysis has potential to assess the engagement of respiratory regulatory network in critically ill patients with sepsis. To assess the complexity of physiological time‐series, a variety of entropy measures have been developed. Sample entropy depicts the irregularity of a signal by estimating the likelihood of repetition of a pattern in physiological time‐series (Richman & Moorman, 2000). A lower value of sample entropy reflects a higher degree of regularity of the physiological signal. However, the higher value of sample entropy cannot distinguish a totally random process from a complex time‐series. To address this ambiguity, Costa et al., extended sample entropy analysis by measuring the changes in entropy at different scales (resolutions) of the original signal. This method, known as multiscale entropy, enables researchers to distinguish between complex signals (e.g., healthy physiological process) and a random process (e.g., white noise) (Costa et al., 2002). Complexity in this context refers to structurally rich and fractal‐like fluctuations reflecting information processing in the physiological system (Goldberger, 1996). When physiological systems become less complex, their information content is reduced which makes them more rigid, less controllable, and less adaptable to the everchanging internal and external environment (Goldberger, 1996; Mazloom et al., 2014; Shirazi et al., 2013). This de‐complexification hypothesis is applicable to many disease states such as sepsis. In fact, sepsis is marked by less complexity of physiological signals (e.g., heart rate and body temperature) than those seen under healthy condition (Gholami et al., 2012; Goldberger, 1996; Lake et al., 2002; Papaioannou et al., 2012, 2013).

SpO_2_ time‐series are complex and fractal‐like specially during hypoxia in healthy individuals (Al Rajeh et al., 2021; Bhogal & Mani, 2017; Costello et al., 2020). Entropy measures have been used in analysis of SpO_2_ time‐series (Al Rajeh et al., 2021; Bhogal & Mani, 2017; Costello et al., 2020; Jiang et al., 2021) and have potential to assess de‐complexification of oxygen saturation signals in critically ill patients. Our objective was to investigate if SpO_2_ entropy analysis can predict survival in sepsis while remaining independent of other measures such as SOFA score, ventilation status, or age. This report aims to highlight the potential for use of oxygen saturation variability in predicting survival in intensive care.

## 2 MATERIALS AND METHODS

### 2.1 Participants

This study is a retrospective cohort study using the Waveform Database Matched Subset of the MIMIC‐III Clinical Database (Johnson et al., 2016). *Inclusion criteria*: Patients over 18 years of age, with a single ICU stay and waveform records in the Waveform Database, who met the Sepsis‐3 criteria on admission (an increase in SOFA score of ≥2 points and suspicion of infection) (Singer et al., 2016). To extract clinical data, a SQL script was adopted from Johnson et al., (Johnson et al., 2018) to replicate the Sepsis‐3 task force criteria. Suspected infection was defined as the acquisition of a body fluid culture temporally contiguous to administration of antibiotics as described before (Johnson et al., 2018). To ensure noise‐free data for this study, patients were only included if their waveform record contained at least 30 min continuous and simultaneous time‐series data for SpO_2_ and respiratory rate. Noise‐free data were defined as having a valid time‐stamped value for each second in the waveform database (sampling rate = 1 Hz). Thus, included time‐series had no missingness and no imputation was needed. The first 30‐min segment of noise‐free waveform was used for calculation of SpO_2_ variability. A total of 179 records had adequate data when including just the three waveforms and these formed the basis of the final cohort. Matched information was retrieved from the Clinical Database on patient age, sex, SOFA scores, mechanical ventilation, lengths of critical care and hospital stay, and date of death. A 30‐day survival data was missing in 15 patients; therefore, 164 patients were included in the final survival analysis (see Data S1 for enrolment data).

### 2.2 Sample entropy and multiscale entropy

**FIGURE 1:**
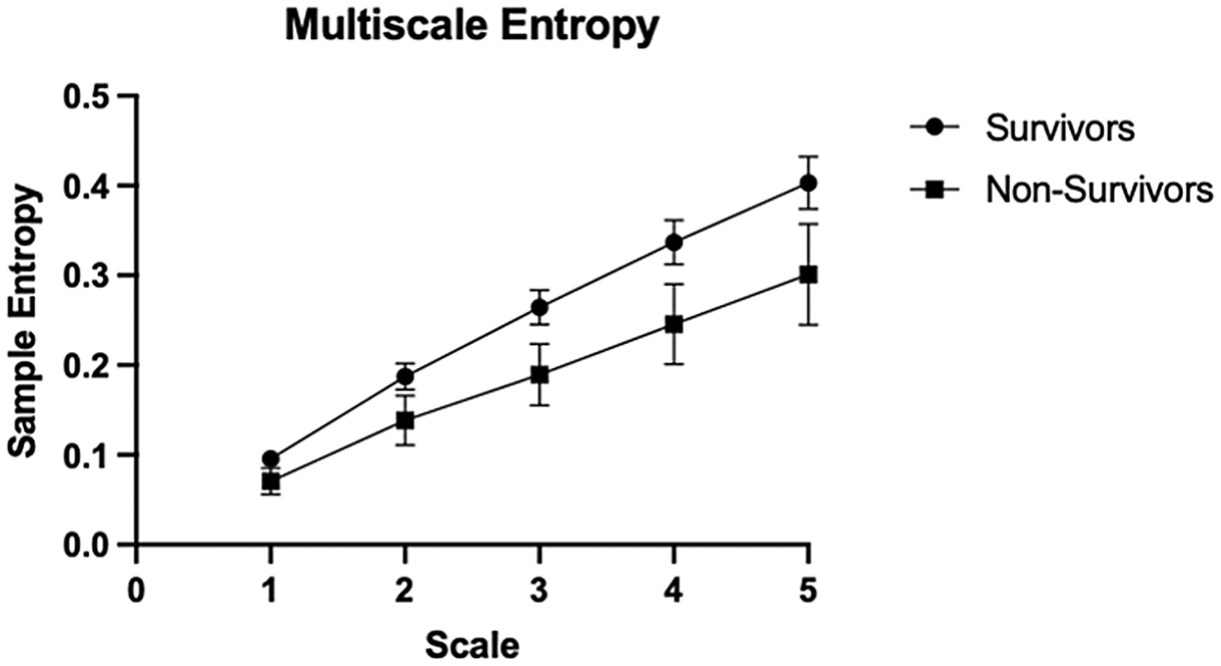
Multiscale entropy of SpO_2_ across 5‐time scales. Error bars represent the standard error mean (SEM).

Sample entropy calculates the probability that epochs of window length m that are similar within a tolerance *r* remain similar at the next point (Richman & Moorman, 2000). Sample entropy was calculated using MATLAB code with a window length, *m*, of 2 and degree of tolerance, *r*, of 0.2 (Bhogal & Mani, 2017; Richman & Moorman, 2000). Multiscale entropy, indicating complexity, was calculated by lowering the resolution of the data by 5‐time scales (1–0.2 Hz) and averaging the sample entropy of survivors versus non‐survivors at each time scale as shown in Figure 1.

### 2.3 Statistical analysis

Data are shown as mean ± SD unless stated otherwise. A multivariate Cox regression was run between the covariates age, SOFA, SpO_2_ mean, SpO_2_ sample entropy, and ventilation status. The ROC curve is employed here to assess the sensitivity and specificity of SpO_2_ mean and sample entropy in classifying the likelihood of patients surviving the ICU stay. Upon analyzing these curves, we decided that combing the SpO_2_ mean and sample entropy in a composite index yielded improved prognostic ability. The composite was calculated by taking the sum of both Cox regression coefficients multiplied by the individual value of the variables. A positive predictive value for the composite score was calculated using cross‐tabulation between those predicted to survive and those who actually survived.

Validation of the composite index was performed by the split‐sample method. A randomly selected sample of 82 patients (training sample) was derived, and the remainder was used as the validation sample (*n* = 82). Cox regression coefficients were recalculated in the training sample, and the regression coefficients were used to calculate the composite in the training sample based on their mean SpO_2_ and SpO_2_ entropy. These calculated composite values in the training sample were used to plot a ROC curve for prediction of survival. This procedure allows for evaluation that the obtained coefficients are substantially independent of the population studied.

## 3 RESULTS

One hundred and thirty patients survived after 30 days follow‐up. Non‐survivors (*n* = 34) had higher age and SOFA scores (Table 1). SpO_2_ mean was slightly higher in survivors than non‐survivors (97.40 ± 2.22 vs. 95.96 ± 6.34, respectively, *p* = 0.033). SpO_2_ entropy was significantly higher in the survivors compared to the non‐survivors (0.107 ± 0.084 and 0.070 ± 0.083, respectively, *p* < 0.05, Cohen's d effect size = 0.441) (see Data S2). This was consistent across different time scales as shown in Figure 1 and Table 1. Two‐way ANOVA showed that there is a statistically significant difference both between scales of measurement as well as outcome groups (*F*_scale_ = 22.48, *p* < 0.0001, and *F*_outcome_ = 11.87, *p* = 0.0006, respectively). Additionally, there is no interaction between scale and group of the multiscale entropy results (*F*_interaction_ = 0.498, *p* = 0.737). Unlike a random time‐series, SpO_2_ fluctuation at larger scales is associated with higher entropy, reflecting nonrandom, complex behavior (Figure 1). Total variability of SpO_2_ time series was assessed using standard deviation, and the values did not differ significantly between survivors and non‐survivors (1.110 ± 0.81 vs. 1.066 ± 1.22, *p* = 0.801). The effect sizes of differences in SpO_2_ mean and variability measures are shown in Data S2.

**TABLE 1:** Comparison of group means for age, SpO_2_‐derived indices, and SOFA. Data are shown as mean ± standard deviation (SD). See Data S2 for Cohen's d effect sizes.

Variables	Survivors	Non‐survivors	*p*‐value
Age (year)	65.265 ± 18.02	75.331 ± 12.44	0.003
SpO_2_ mean (%)	97.40 ± 2.22	95.96 ± 6.34	0.033
Spo_2_ standard deviation	1.110 ± 0.81	1.066 ± 1.22	0.801
SpO_2_ entropy (Scale 1)	0.107 ± 0.08	0.070 ± 0.08	0.030
SpO_2_ entropy (Scale 2)	0.210 ± 0.16	0.138 ± 0.16	0.028
SpO_2_ entropy (Scale 3)	0.298 ± 0.22	0.189 ± 0.19	0.008
SpO_2_ entropy (Scale 4)	0.378 ± 0.28	0.245 ± 0.25	0.012
SpO_2_ entropy (Scale 5)	0.453 ± 0.33	0.301 ± 0.32	0.021
SOFA	4.100 ± 2.26	6.820 ± 4.15	<0.001

Table 2 displays the multivariate Cox regression analysis between age, SOFA, SpO_2_ mean and sample entropy, and ventilation status. All covariates have a statistically significant (*p* < 0.01), independent impact on the outcome of mortality. A negative slope (*β*) indicates a protective measure. In this case, increases in SpO_2_ mean and sample entropy are protective. As expected, age, SOFA, and ventilation status were strong predictors of mortality (*p* < 0.001).

**TABLE 2:** Independence of SpO_2_ measures from SOFA, age, and ventilation status in predicting mortality with Cox multivariate regression analysis.

Covariates	*β*	SE	Hazard ratio	95.0% CI for Hazard ratio		*p*‐value
				Lower	Upper	
Age	0.047	0.014	1.048	1.020	1.077	0.001
SOFA	0.191	0.051	1.211	1.096	1.338	<0.001
SpO_2_ mean	−0.589	0.168	0.555	0.399	0.771	<0.001
SpO_2_ entropy	−0.798	0.292	0.450	0.254	0.798	0.006
Ventilation status	1.452	0.406	4.272	1.929	9.458	<0.001

*Note*: The interaction of SpO_2_ mean and entropy was assessed during multivariate regression analysis, which showed no significant interaction. To make interpretation of hazard ratios of SpO_2_ mean and entropy comparable, the scales of SpO_2_ mean and entropy were standardized in the Cox model using Z transformation.

**FIGURE 2:**
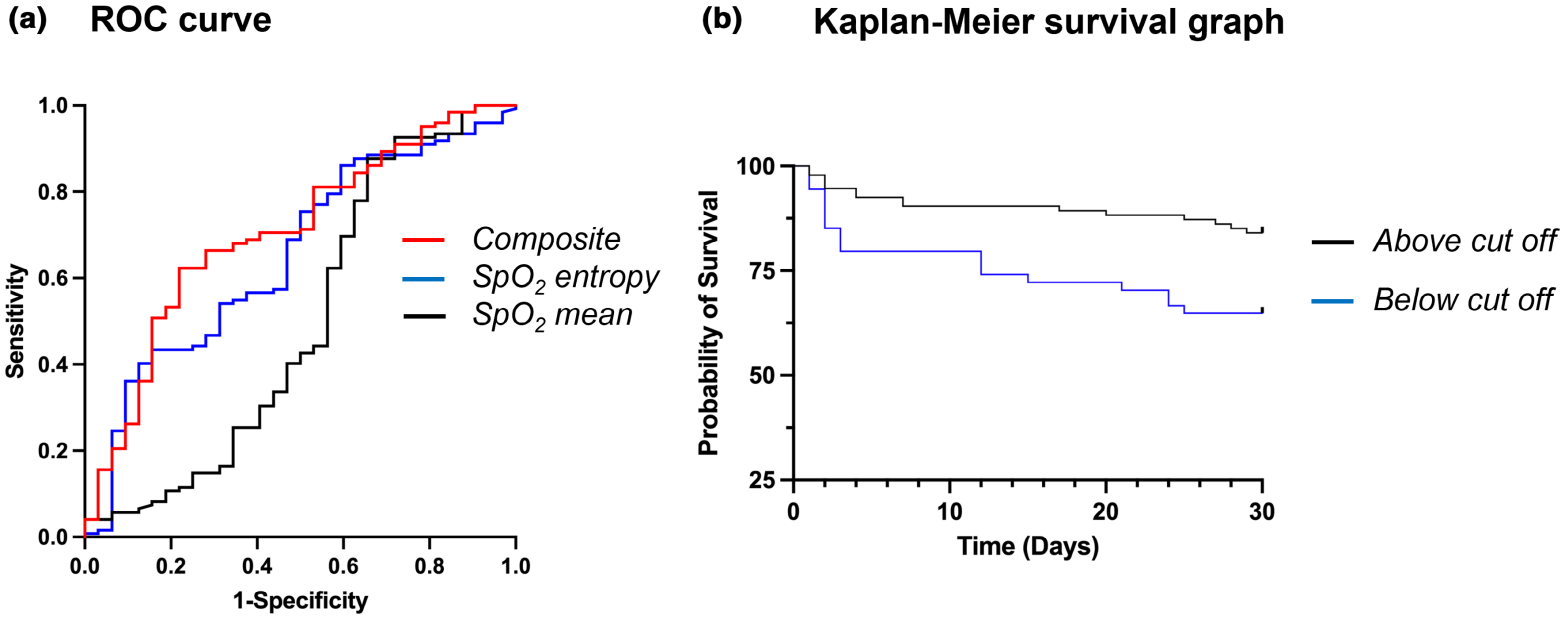
(a) ROC curve for classifying survival in critically ill patients with sepsis based on SpO_2_ mean (AUC = 0.498), SpO_2_ entropy (AUC = 0.654), and composite SpO_2_ mean and entropy (AUC = 0.705). (b) Survival analysis of patients above and below optimum composite cut off obtained from ROC curve.

ROC curve analysis (Figure 2a) shows SpO_2_ sample entropy as a decent indicator of survival, with an area under the curve (AUC) of 0.654 (*p* = 0.007). However, when combined with SpO_2_ mean, the classifying power is increased (AUC = 0.705, *p* < 0.001) (Table 3). Taking the Youden Index, a Kaplan–Meier graph was plotted to visualize the value of SpO_2_ entropy and mean in predicting survival (Figure 2b). As shown in the figure, patients above the composite index exhibited an 84.043% survival rate versus those below the composite score who showed only a 64.815% survival rate (Log‐rank test *p* = 0.0027). A positive predictive value for the composite was calculated for survival analysis, and 87.5% of those assessed to survive, did survive. This is approximately 8% higher than the probability of survival in this cohort without consideration of any physiological signal (79.2%). We were also wondering about ROC performance for the other scales of SpO_2_ entropy. As shown in Data S3, the AUC of SpO_2_ entropy were significantly higher than 0.5 at all scales. However, scale 1 showed the highest AUC, and therefore, the rest of survival analyses were carried out using SpO_2_ entropy at scale 1.

**TABLE 3:** The AUC of ROC analysis for clinical and physiological indices assessed in this study.

Variables	AUC (95% CI)	*p*‐value
SpO_2_ mean	0.498 (0.373–0.625)	0.970
SpO_2_ entropy	0.654 (0.548–0.760)	0.007
Composite SpO_2_ mean and entropy	0.705 (0.604–0.806)	<0.001
SOFA	0.707 (0.603–0.810)	<0.001
Composite SpO_2_ entropy and SOFA	0.793 (0.705–0.881)	<0.001

*Note*: *p*‐values were calculated to test the null hypothesis of AUC = 0.5.

**FIGURE 3:**
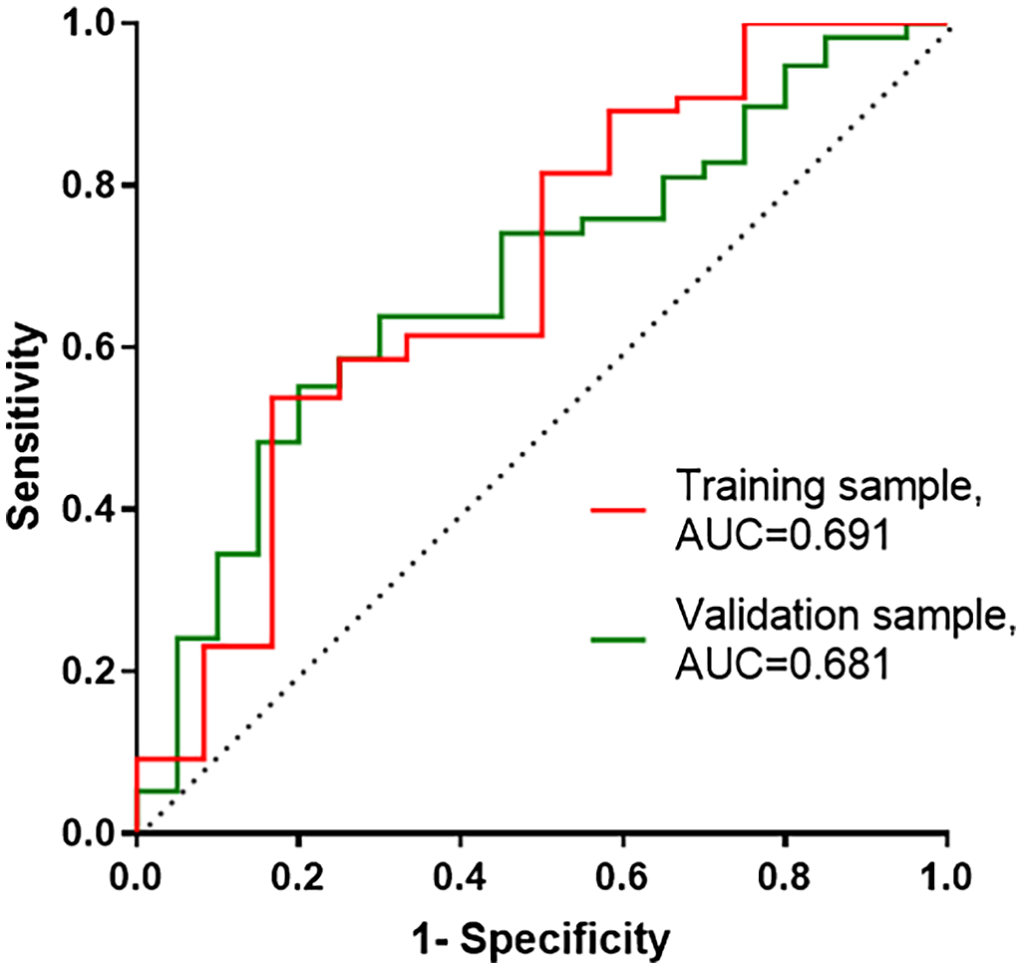
ROC curves for classifying survival in training and validation samples. Composite SpO_2_ mean and entropy index for each patient in the validation sample was calculated based on Cox regression coefficients of the training sample. AUC of both samples are significantly different from a random classifier (AUC = 0.5), *p* = .036 and *p* = .016 for the training and validation samples, respectively.

The validity of the composite model in prediction of survival was assessed using a split‐sample technique. The composite index (SpO_2_ mean and entropy combined) was recalculated in a training sample of 82 randomly selected patients. The individual composite was calculated for the validation sample comprising the remainder 82 patients. The ROC curves for the training sample and validation sample are depicted in Figure 3. AUC was similar for both training and validation samples (0.691 ± 0.089 vs. 0.681 ± 0.067, respectively). Both samples exhibited an AUC which is significantly higher than a random classifier AUC (*p* < 0.05).

The effect of ventilation status: Although Cox regression reports ventilation status as an independent predictor of mortality in this set of patients, additional calculations were conducted investigating the characteristics of ventilated and non‐ventilated patients. The comparison of group statistics shows significant differences in SpO_2_ sample entropy at all scales, with those non‐ventilated exhibiting significantly higher levels (Data S4). Further, a multivariate Cox regression was run on non‐ventilated patients alone, and SpO_2_ entropy was again an independent predictor of mortality (Data S5).

## 4 DISCUSSION

Oxygen saturation variability has rarely been applied in assessment of patients' prognosis or disease severity (Garde et al., 2016). This is surprising, as SpO_2_ signal can be measured easily in both inpatient and outpatient settings, and there are well‐established algorithms to estimate entropy of physiological time‐series. Our results demonstrated for the first time that SpO_2_ entropy can be a predictor of mortality in critically ill patients with sepsis. The sample entropy of SpO_2_ over multiple time scales is significantly lower in those who did not survive in the ICU. This finding is consistent with previous studies indicating that SpO_2_ fluctuations can give insight into the autonomic control of the cardiorespiratory system, and moreover, increased SpO_2_ variability is resultant of physiological systems engaging in a transfer of information (Jiang et al., 2021). Further, the variability of other cardiorespiratory indices such as respiratory rate does not offer any predictive value to ICU survival in this cohort (see Data S6 and S7). This further demonstrates the importance of SpO_2_ variability as an indicator of system engagement. Pulse oximetry is already measured for intensive care patients, and this time‐series analysis can easily be implemented with a simple computer program or smart device. While SpO_2_ mean has an inadequate sensitivity and specificity for prediction of survival on its own, when combined with SpO_2_ sample entropy, the two have quite a substantial predictive power. Multivariate analysis confirmed that SpO_2_ sample entropy is an independent predictor of mortality, and further, when combined with mean SpO_2_, the composite has an AUC of 0.70. This is comparable to the SOFA score with an AUC of 0.71 in our study group. Furthermore, addition of SpO_2_ entropy has potential to increase the prognostic value of SOFA, as the AUC of SOFA‐SpO_2_ entropy is higher (0.79) than SOFA alone (Table 3).

We were wary that a patient's ventilation status during SpO_2_ recording could cause a significant effect on SpO_2_ pattern of fluctuation and hence, its entropy. However, as a supplemental investigation, we analyzed the same trends on patients who were non‐ventilated and found similar results (Data S4 and S5). Despite having a significantly greater average SpO_2_ mean, those who were ventilated had significantly lower SpO_2_ sample entropy and were less likely to survive their ICU stay (non‐ventilated 89% vs ventilated 63%, Log‐rank test *p* < 0.0001). This goes along with the initial multivariate Cox regression, showing that the prognostic effect of SpO_2_ entropy is independent of the ventilation status (Table 2). Moreover, higher average SpO_2_ mean in ventilated patients might indicate that the target oxygen saturation in ventilated patients may have been higher than the optimum physiological level. There are recent concerns on the impact that supraphysiological oxygen supplementation could have on patient outcome. A meta‐analysis by Chu et al. (2018) report that in over 16,000 critically ill patients, those receiving more conservative oxygenation had a higher survival rate compared to the more liberally oxygenated patients. Further, the liberally ventilated group showed higher mortality and did not show improvement in other patient‐important outcomes. Given that there is not a conclusive benchmark for the target oxygen saturation in critically ill patients, further research into the relationship between SpO_2_ entropy and mean in ventilated critically ill patients may have potential to provide information for optimum oxygen supplementation in future.

Interestingly, in mechanically ventilated patients, SpO_2_ time‐series shows subtle fluctuations which might be linked to other feedback loops within the patient control system. Details of ventilator setup was not available in the MIMIC III dataset, and we could not include them in our analysis. However, to shed light on the effect of respiratory pattern on SpO_2_ fluctuations, we calculated the reciprocal interaction between respiratory rate (RR) and SpO_2_ time‐series in mechanically ventilated and spontaneously breading patients. For this analysis, we used the concept of transfer entropy (TE) to measure the exchange of information from a physiological signal (e.g., RR) to another (e.g., SpO_2_) and vice versa (Jiang et al., 2021; Schreiber, 2000). Our results showed that both TE (RR ➔ SpO_2_) and TE (SpO_2_ ➔ RR) is significantly lower in mechanically ventilated patients compared with nonmechanically ventilated participants (Data S8). This is an expected finding as mechanical ventilation minimizes the spontaneous physiological feedback loops in control of respiratory pattern. In addition, this type of analysis shows that the concept of entropy (e.g., sample entropy or transfer entropy) can be used to assess the engagement of respiratory control system. TE (RR ➔ SpO_2_) was small but measurable for many mechanically ventilated patients. This also reflects the presence of other feedback loops which requires more comprehensive network analysis as reported by Jiang et al. (Jiang et al., 2021).

There are multiple analytical methods for assessment of variability of physiological time‐series. Entropy is attractive, as it is linked with the concept of information theory (Bhogal & Mani, 2017; Costello et al., 2020; Jiang et al., 2021). Simpler methods such as standard deviation can be applied to measure total SpO_2_ variability (Garde et al., 2016; Moss et al., 2016); thus, we wondered if the standard deviation of SpO_2_ had any predictive value in our cohort. Our results showed that standard deviation of SpO_2_ time‐series is not significantly different in survivors and non‐survivors (Table 1). Furthermore, Cox regression analysis did not show any prognostic value for this measure of total variability (hazard ratio = 0.985, *p* = 0.943). This goes along with a previous report that SpO_2_ entropy and not its standard deviation correlates with the intensity of dyspnea in experimental hypoxia (Costello et al., 2020). In general, standard deviation of time‐series is not an accurate measure of fluctuations in complex systems, and our findings confirm that the measures of complexity (e.g., sample entropy or multiscale entropy) rather than total variability of oxygen saturation time‐series exhibits a prognostic value in critically ill patients with sepsis.

According to this report, reduced entropy of SpO_2_ time‐series is associated with poor prognosis in patients with sepsis independently from potential confounders such as age, SOFA, and ventilation status. These findings go along with the concept of de‐complexification in disease states outlined by Goldberger and colleagues (Goldberger, 1996, 2006). Reduced structural richness of a physiological time‐series may indicate less information content which makes the system less adaptable/controllable to internal and external challenges. Reduced complexity of cardiac time‐series as well as body temperature have already been reported in non‐survivors with sepsis (de Castilho et al., 2018; Papaioannou et al., 2012, 2013). Future studies can extend this type of computational analysis by including network analysis of parallel physiological variables for providing pathophysiologic insight as well as personalized physio‐markers for prognostication in critically ill patients (Moorman et al., 2016; Zhang et al., 2022).

A noteworthy limitation of this study is the sample size. However, for this initial investigation, we were more concerned with obtaining noise‐free data before expanding the analysis to a larger group. Further, the majority of ICU signals are not always so clean, and this could be an obstacle for further investigations. This point also brings up the question of recording length. We used 30‐min length signals, as previous reports have shown that SpO_2_ entropy calculated from 30‐min time‐series provide information about the integrity of cardiorespiratory control during hypoxia (Jiang et al., 2021). A next step could be calculating the optimal length of recording which would still have sufficient prognostic value. While this is a promising prognostic tool, more work needs to be done before translation into a clinical setting. This analysis was conducted only within the context of sepsis, but the results are promising enough to warrant investigation in all patients in critical care settings as well as in diseases associated with respiratory distress. The source of ICU data in our study is the MIMIC III dataset and was collected between 2001 and 2012 at the critical care units of the Beth Israel Deaconess Medical Center in North America. Therefore, our results may not be generalizable to other ICU settings. In our sample, ethnicity was not a predictor of 30‐day mortality (data not shown), however, multiple factors may influence the cause of sepsis and intensive care mortality which are not investigated in this report. There are potentially other limitations in this study. Detailed hemodynamics data (e.g., blood pressure time‐series) and ventilation parameters were not available for inclusion in this analysis. We are also wary that there is a limit in accuracy of pulse oximeters in estimation of oxygen saturation during hypoxia (Thrush & Hodges, 1994). Pulse oximeters might show inaccuracy in measurement of true oxygen saturation (SaO_2_) when it is below 90%. Within our dataset, all patients (expect one) had a mean SpO_2_ higher than 90% (mean SpO_2_ was 97.40 ± 2.22 in survivors and 95.96 ± 6.34 in non‐survivors). Thus, the range of fluctuations of SpO_2_ is below the level that raises concern within the context of pulse oximeters accuracy according to previous reports (Thrush & Hodges, 1994). This will reduce the likelihood of measurement bias in this report. However, there is a possibility that inaccuracy of pulse oximeters can affect the pattern pf SpO_2_ time‐series and hence cofound our results. This limitation needs to be addressed in future investigations.

## 5 CONCLUSION

This study demonstrated an increase in survival for critically ill patients with sepsis who exhibit a higher sample entropy of the SpO_2_ signal. Multivariate analysis confirmed that SpO_2_ sample entropy is an independent predictor of mortality, and further, when combined with SpO_2_ mean, the composite measure has an AUC of 0.705. This is comparable to the current SOFA score and requires fewer tests. Taking SpO_2_ measurements already occurs in the ICU, thus making entropy analysis a simple practice to incorporate. However, application of this method in healthcare warrants further investigations in larger studies.

## AUTHORS' CONTRIBUTION

Ali R. Mani, Watjana Lilaonitkul, and Margaret Gheorghita conceived the study and formulated the concept of oxygen saturation variability analysis in sepsis. Matthew Wikner, Anika Cawthorn, Patrick Rockenschaub, Ferran Gonzalez Hernandez, Nel Swanepoel, Tope Oyelade, and Watjana Lilaonitkul extracted data from MIMIC III database. Margaret Gheorghita, Matthew Wikner, Kristof Nemeth, and Ali R Mani performed computational analysis of physiological time‐series and statistical analysis. Margaret Gheorghita wrote the first manuscript draft, and all authors revised it for important intellectual content. All authors read and approved the final manuscript.

## FUNDING INFORMATION

No funding information provided.

## CONFLICT OF INTEREST

The authors declare that the research was conducted in the absence of any commercial or financial relationships that could be construed as a potential conflict of interest.

## Ethics statement

MIMIC‐III is publicly available and made accessible to researchers under a data use agreement. The data has been deidentified in accordance with Health Insurance Portability and Accountability Act (HIPAA) standards and the project approved by the Institutional Review Boards of Beth Israel Deaconess Medical Center and the Massachusetts Institute of Technology (IRB protocol nos. 2001P001699/14 and 0403000206, respectively; Johnson et al., 2016). Requirement for individual patient consent was waived because the project did not impact clinical care and all protected health information was deidentified. The authors involved in data extraction completed mandatory online ethics training at MIT and were credentialled on 28 Jul 2022 (ID 10304625). The study was exempt from the need for specific permission by the UCL Research Ethics Committee (see https://ethics.grad.ucl.ac.uk/exemptions.php).

## Supporting information


Data S1‐S8

## References

[phy215546-bib-0001] Al Rajeh, A. , Bhogal, A. S. , Zhang, Y. , Costello, J. T. , Hurst, J. R. , & Mani, A. R. (2021). Application of oxygen saturation variability analysis for the detection of exacerbation in individuals with COPD: A proof‐of‐concept study. Physiological Reports, 9(23), e15132. 10.14814/phy2.15132 34851045 PMC8634631

[phy215546-bib-0002] Asada, T. , Aoki, Y. , Sugiyama, T. , Yamamoto, M. , Ishii, T. , Kitsuta, Y. , Nakajima, S. , & Yahagi, N. (2016). Organ system network disruption in nonsurvivors of critically ill patients. Critical Care Medicine, 44(1), 83–90. 10.1097/CCM.0000000000001354 26496455

[phy215546-bib-0003] Bhogal, A. , & Mani, A. R. (2017). Pattern analysis of oxygen saturation variability in healthy individuals: Entropy of pulse oximetry signals carries information about mean oxygen saturation. Frontiers in Physiology, 8, 555. 10.3389/fphys.2017.00555 28824451 PMC5539125

[phy215546-bib-0004] Chu, D. K. , Kim, L. H. , Young, P. J. , Zamiri, N. , Almenawer, S. A. , Jaeschke, R. , Szczeklik, W. , Schünemann, H. J. , Neary, J. D. , & Alhazzani, W. (2018). Mortality and morbidity in acutely ill adults treated with liberal versus conservative oxygen therapy (IOTA): A systematic review and meta‐analysis. Lancet, 391(10131), 1693–1705. 10.1016/S0140-6736(18)30479-3 29726345

[phy215546-bib-0005] Costa, M. , Goldberger, A. L. , & Peng, C. K. (2002). Multiscale entropy analysis of complex physiologic time series. Physical Review Letters, 89(6), 068102. 10.1103/PhysRevLett.89.068102 12190613

[phy215546-bib-0006] Costello, J. T. , Bhogal, A. S. , Williams, T. B. , Bekoe, R. , Sabir, A. , Tipton, M. J. , Corbett, J. , & Mani, A. R. (2020). Effects of normobaric hypoxia on oxygen saturation variability. High Altitude Medicine and Biology, 21(1), 76–83. 10.1089/ham.2019.0092 32069121

[phy215546-bib-0007] de Castilho, F. M. , Ribeiro, A. L. P. , Nobre, V. , Barros, G. , & de Sousa, M. R. (2018). Heart rate variability as predictor of mortality in sepsis: A systematic review. PLoS One, 13(9), e0203487. 30204803 10.1371/journal.pone.0203487 PMC6133362

[phy215546-bib-0008] Garde, A. , Zhou, G. , Raihana, S. , Dunsmuir, D. , Karlen, W. , Dekhordi, P. , Huda, T. , El Arifeen, S. , Larson, C. , Kissoon, N. , & Dumont, G. A. (2016). Respiratory rate and pulse oximetry derived information as predictors of hospital admission in young children in Bangladesh: A prospective observational study. BMJ Open, 6(8), e011094. 10.1136/bmjopen-2016-011094 PMC5013424 27534987

[phy215546-bib-0009] Gholami, M. , Mazaheri, P. , Mohamadi, A. , Dehpour, T. , Safari, F. , Hajizadeh, S. , Moore, K. P. , & Mani, A. R. (2012). Endotoxemia is associated with partial uncoupling of cardiac pacemaker from cholinergic neural control in rats. Shock, 37(2), 219–227. 10.1097/SHK.0b013e318240b4be 22249221

[phy215546-bib-0010] Goldberger, A. L. (1996). Non‐linear dynamics for clinicians: Chaos theory, fractals, and complexity at the bedside. Lancet, 347(9011), 1312–1314. 10.1016/s0140-6736(96)90948-4 8622511

[phy215546-bib-0011] Goldberger, A. L. (2006). Giles f. Filley lecture. Complex systems. Proceedings of the American Thoracic Society, 3(6), 467–471. 10.1513/pats.200603-028MS 16921107 PMC2647633

[phy215546-bib-0012] Jiang, Y. , Costello, J. T. , Williams, T. B. , Panyapiean, N. , Bhogal, A. S. , Tipton, M. J. , Corbett, J. , & Mani, A. R. (2021). A network physiology approach to oxygen saturation variability during normobaric hypoxia. Experimental Physiology, 106(1), 151–159. 10.1113/EP088755 32643311

[phy215546-bib-0013] Johnson, A. E. , Aboab, J. , Raffa, J. D. , Pollard, T. J. , Deliberato, R. O. , Celi, L. A. , & Stone, D. J. (2018). A comparative analysis of sepsis identification methods in an electronic database. Critical Care Medicine, 46(4), 494–499. 10.1097/CCM.0000000000002965 29303796 PMC5851804

[phy215546-bib-0014] Johnson, A. E. , Pollard, T. J. , Shen, L. , Lehman, L. W. H. , Feng, M. , Ghassemi, M. , Moody, B. , Szolovits, P. , Anthony Celi, L. , & Mark, R. G. (2016). MIMIC‐III, a freely accessible critical care database. Scientific Data, 3, 160035. 10.1038/sdata.2016.35 27219127 PMC4878278

[phy215546-bib-0015] Lake, D. E. , Richman, J. S. , Griffin, M. P. , & Moorman, J. R. (2002). Sample entropy analysis of neonatal heart rate variability. American Journal of Physiology. Regulatory, Integrative and Comparative Physiology, 283(3), R789–R797. 10.1152/ajpregu.00069.2002 12185014

[phy215546-bib-0016] Mazloom, R. , Shirazi, A. H. , Hajizadeh, S. , Dehpour, A. R. , & Mani, A. R. (2014). The effect of endotoxin on the controllability of cardiac rhythm in rats. Physiological Measurement, 35(3), 339–349. 10.1088/0967-3334/35/3/339 24480859

[phy215546-bib-0017] Moorman, J. R. , Lake, D. E. , & Ivanov, P. C. (2016). Early detection of sepsis—a role for network physiology? Critical Care Medicine, 44(5), e312–e313. 10.1097/CCM.0000000000001548 27083036

[phy215546-bib-0018] Moss, T. J. , Lake, D. E. , Calland, J. F. , Enfield, K. B. , Delos, J. B. , Fairchild, K. D. , & Moorman, J. R. (2016). Signatures of subacute potentially catastrophic illness in the ICU: Model development and validation. Critical Care Medicine, 44(9), 1639–1648. 10.1097/CCM.0000000000001738 27452809 PMC4987175

[phy215546-bib-0019] Papaioannou, V. E. , Chouvarda, I. G. , Maglaveras, N. K. , Baltopoulos, G. I. , & Pneumatikos, I. A. (2013). Temperature multiscale entropy analysis: A promising marker for early prediction of mortality in septic patients. Physiological Measurement, 34(11), 1449–1466. 10.1088/0967-3334/34/11/1449 24149496

[phy215546-bib-0020] Papaioannou, V. E. , Chouvarda, I. G. , Maglaveras, N. K. , & Pneumatikos, I. A. (2012). Temperature variability analysis using wavelets and multiscale entropy in patients with systemic inflammatory response syndrome, sepsis, and septic shock. Critical Care, 16(2), R51. 10.1186/cc11255 22424316 PMC3681376

[phy215546-bib-0021] Richman, J. , & Moorman, J. (2000). Physiological time‐series analysis using approximate and sample entropy. American Journal of Physiology – Heart and Circulatory Physiology, 278(6), H2039–H2049. 10.1152/ajpheart.2000.278.6.h2039 10843903

[phy215546-bib-0022] Roe, P. G. , & Jones, J. G. (1993). Causes of oxyhaemoglobin saturation instability in the postoperative period. British Journal of Anaesthesiology, 71(4), 481–487. 10.1093/bja/71.4.481 8260293

[phy215546-bib-0023] Rudd, K. E. , Johnson, S. C. , Agesa, K. M. , Shackelford, K. A. , Tsoi, D. , Kievlan, D. R. , Colombara, D. V. , Ikuta, K. S. , Kissoon, N. , Finfer, S. , Fleischmann‐Struzek, C. , Machado, F. R. , Reinhart, K. K. , Rowan, K. , Seymour, C. W. , Watson, R. S. , West, T. E. , Marinho, F. , Hay, S. I. , … Naghavi, M. (2020). Global, regional, and national sepsis incidence and mortality, 1990–2017: Analysis for the global burden of disease study. Lancet, 395(10219), 200–211. 10.1016/S0140-6736(19)32989-7 31954465 PMC6970225

[phy215546-bib-0024] Schreiber, T. (2000). Measuring information transfer. Physical Review Letters, 85(2), 461–464. 10.1103/PhysRevLett.85.461 10991308

[phy215546-bib-0025] Shirazi, A. H. , Raoufy, M. R. , Ebadi, H. , De Rui, M. , Schiff, S. , Mazloom, R. , Hajizadeh, S. , Gharibzadeh, S. , Dehpour, A. R. , Amodio, P. , Jafari, G. R. , Montagnese, S. , & Mani, A. R. (2013). Quantifying memory in complex physiological time‐series. PLoS One, 8(9), e72854. 10.1371/journal.pone.0072854 24039811 PMC3764113

[phy215546-bib-0026] Singer, M. , Deutschman, C. S. , Seymour, C. W. , Shankar‐Hari, M. , Annane, D. , Bauer, M. , Bellomo, R. , Bernard, G. R. , Chiche, J. D. , Coopersmith, C. M. , & Hotchkiss, R. S. (2016). The third international consensus definitions for sepsis and septic shock (Sepsis‐3). Journal of the American Medical Association, 315(8), 801–810. 10.1001/jama.2016.0287 26903338 PMC4968574

[phy215546-bib-0027] Thrush, D. , & Hodges, M. R. (1994). Accuracy of pulse oximetry during hypoxemia. Southern Medical Journal, 87(4), 518–521. 10.1097/00007611-199404000-00019 8153783

[phy215546-bib-0028] Tuchschmidt, J. , Oblitas, D. , & Fried, J. (1991). Oxygen consumption in sepsis and septic shock. Critical Care Medicine, 19(5), 664–671. 10.1097/00003246-199105000-00013 2026029

[phy215546-bib-0029] Zhang, H. , Oyelade, T. , Moore, K. P. , Montagnese, S. , & Mani, A. R. (2022). Prognosis and survival modelling in cirrhosis using parenclitic networks. Frontiers in Network Physiology, 2, 833119. 10.3389/fnetp.2022.833119 PMC10013061 36926100

