# Supplementary material for: Reduced oxygen saturation entropy is associated with poor prognosis in critically ill patients with sepsis
Source: Physiol Rep. 2022 Dec 21;10(24):e15546. doi: 10.14814/phy2.15546 (PMC9768724; doi:10.14814/phy2.15546)
Supplement: Supplementary file 1 — Data S1‐S8 [file PHY2-10-e15546-s001.docx]

**Supplements**

**Supplement S1**. Patient enrolment flow diagram. HR: heart rate; RESP: respiratory rate; SpO_2_: oxygen saturation.

Single ICU stay and sepsis-3 criteria met in Clinical Database

(n=4540)

+ only one record in Waveform Database

(n=374)

+ 30 minutes of continuous SpO_2_, HR and RES time-series data

(n=179)

+ Clinical and Waveform Database dates match

(n=164)

**Supplement S2.** Cohen’s d effect sizes during comparison of survivor and non-survivor groups for age, SpO_2_-derived indices, and SOFA score.

|  | **Cohen’s d** | **95% Confidence Interval** |
| --- | --- | --- |
| **Age (year)** | -0.591 | -0.973 – -0.207 |
| **SpO_2_ mean (%)** | 0.414 | 0.033 – 0.793 |
| **SpO_2_ entropy (Scale 1)** | 0.441 | 0.48 – 0.833 |
| **SpO_2_ entropy (Scale 2)** | 0.442 | 0.049 – 0.833 |
| **SpO_2_ entropy (Scale 3)** | 0.507 | 0.113 – 0.900 |
| **SpO_2_ entropy (Scale 4)** | 0.485 | 0.092 – 0.878 |
| **SpO_2_ entropy (Scale 5)** | 0.465 | 0.072 – 0.857 |
| **SOFA** | -0.990 | -1.381 – -0.596 |
| **Composite SpO_2_ entropy and SOFA** | 0.804 | 0.404 – 1.203 |

**Supplement S3. A.** ROC curve for classifying survival in critically ill patients with sepsis based on SpO_2_ entropy at difference scales (multiscale entropy analysis). **(B)** The AUC of ROC analysis for prediction of survival based on SpO2 entropy at different scales. P-values were calculated to test the null hypothesis of AUC=0.5.

**A.**


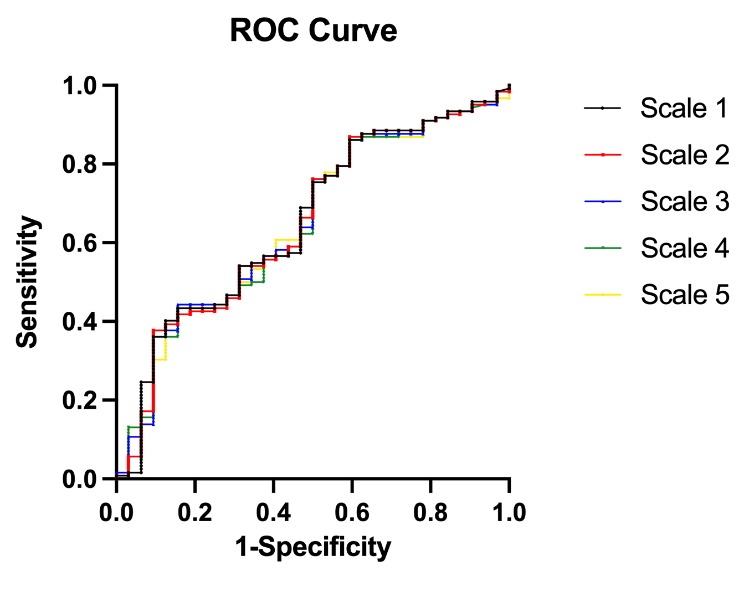


| **B.** | **AUC (95 % CI)** | **P-value** |
| --- | --- | --- |
| **SpO_2_ entropy (scale 1)** | 0.654 (0.548 - 0.760) | 0.007 |
| **SpO_2_ entropy (scale 2)** | 0.651 (0.544 - 0.758) | 0.009 |
| **SpO_2_ entropy (scale 3)** | 0.651 (0.545 - 0.757) | 0.009 |
| **SpO_2_ entropy (scale 4)** | 0.647 (0.541 - 0.753) | 0.010 |
| **SpO_2_ entropy (scale 5)** | 0.649 (0.543 - 0.755) | 0.009 |

**Supplement S4.** Comparison of ventilated and non-ventilated group means. Data are shown as mean ± SD.

|  | **Ventilated** | **Non-Ventilated** | **P-value** |
| --- | --- | --- | --- |
| **Age (year)** | 63.856 ± 16.08 | 68.674 ± 17.85 | 0.101 |
| **SpO_2_ mean (%)** | 98.269 ± 2.75 | 96.657 ± 3.68 | 0.003 |
| **Spo_2_ standard deviation** | 0.679 ± 0.65 | 1.261 ± 0.94 | <0.001 |
| **SpO_2_ entropy (Scale 1)** | 0.050 ± 0.05 | 0.117 ± 0.07 | <0.001 |
| **SpO_2_ entropy (Scale 2)** | 0.098 ± 0.11 | 0.229 ± 0.17 | <0.001 |
| **SpO_2_ entropy (Scale 3)** | 0.142 ± 0.16 | 0.323 ± 0.22 | <0.001 |
| **SpO_2_ entropy (Scale 4)** | 0.183 ± 0.20 | 0.410 ± 0.28 | <0.001 |
| **SpO_2_ entropy (Scale 5)** | 0.218 ± 0.24 | 0.493 ± 0.33 | <0.001 |
| **SOFA** | 5.16 ± 3.00 | 4.48 ± 2.93 | 0.199 |

**Supplement S5.** Multivariate Analysis for non-ventilated patients (n=114). To make interpretation of hazard ratios of SpO_2_ mean and entropy comparable, the scales of SpO_2_ mean and entropy were standardized in the Cox model using Z transformation.

|  | **β** | **SE** | **Hazard Ratio** | **95 % CI for Hazard Ratio** | | **P-value** |
| --- | --- | --- | --- | --- | --- | --- |
|  |  |  |  | **Lower** | **Upper** |  |
| **Age** | 0.048 | 0.021 | 1.049 | 1.006 | 1.093 | 0.025 |
| **SOFA** | 0.197 | 0.062 | 1.217 | 1.079 | 1.373 | 0.001 |
| **SpO_2_ mean** | -0.488 | 0.198 | 0.614 | 0.417 | 0.905 | 0.014 |
| **SpO_2_ entropy** | -0.762 | 0.352 | 0.467 | 0.234 | 0.930 | 0.030 |

**Supplement S6.** Comparison of respiration rate (RR) mean and variability indices between survivor and non-survivor groups.

|  | **Survivor** | **Non-Survivor** | **P-value** |
| --- | --- | --- | --- |
| **Mean respiration rate (bpm)** | 19.673 ± 4.79 | 21.211 ± 6.02 | 0.175 |
| **Respiration rate standard deviation** | 2.940 ± 1.46 | 2.423 ± 1.47 | 0.073 |
| **RR entropy (Scale 1)** | 0.313 ± 0.18 | 0.252 ± 0.21 | 0.126 |
| **RR entropy (Scale 2)** | 0.558 ± 0.29 | 0.447 ± 0.33 | 0.082 |
| **RR entropy (Scale 3)** | 0.739 ± 0.36 | 0.548 ± 0.37 | 0.009 |
| **RR entropy (Scale 4)** | 0.835 ± 0.37 | 0.660 ± 0.43 | 0.037 |
| **RR entropy (Scale 5)** | 0.942 ± 0.41 | 0.744 ± 0.49 | 0.036 |

**Supplement S7.** Multivariate Cox regression analysis of respiratory rate variability indices for prediction of 30-day mortality

|  | **B** | **SE** | **Hazard Ratio** | **P-value** |
| --- | --- | --- | --- | --- |
| **SOFA** | 0.223 | 0.052 | 1.250 | <0.001 |
| **Mean respiration rate** | 0.063 | 0.033 | 1.065 | 0.059 |
| **RR entropy (Scale 1)** | 6.389 | 5.471 | 595.266 | 0.243 |
| **RR entropy (Scale 2)** | -2.410 | 5.127 | 0.090 | 0.638 |
| **RR entropy (Scale 3)** | -5.747 | 3.270 | 0.003 | 0.079 |
| **RR entropy (Scale 4)** | 1.261 | 2.579 | 3.529 | 0.625 |
| **RR entropy (Scale 5)** | 1.716 | 2.943 | 5.564 | 0.560 |
| **Ventilation Status** | 0.975 | 0.408 | 2.652 | 0.017 |

**Supplement S8.** Comparison of transfer entropy (TE) between respiratory rate (RR) and SpO_2_ time-series in patients with or without mechanical ventilation. TE was calculated as described by Jiang et al., (3).

|  | **Ventilated** | **Non-Ventilated** | **P-value** |
| --- | --- | --- | --- |
| **TE (RR 🡪 SpO_2_)** | 0.101 ± 0.127 | 0.195 ± 0.128 | <0.001 |
| **TE (SpO_2_ 🡪 RR)** | 0.143 ± 0.165 | 0.214 ± 0.167 | 0.016 |
